# Bioinformatics and molecular biology tools for diagnosis, prevention, treatment and prognosis of COVID-19

**DOI:** 10.1016/j.heliyon.2024.e34393

**Published:** 2024-07-11

**Authors:** Débora Dummer Meira, Aléxia Stefani Siqueira Zetum, Matheus Correia Casotti, Danielle Ribeiro Campos da Silva, Bruno Cancian de Araújo, Creuza Rachel Vicente, Daniel de Almeida Duque, Bianca Paulino Campanharo, Fernanda Mariano Garcia, Camilly Victória Campanharo, Carla Carvalho Aguiar, Carolina de Aquino Lapa, Flávio dos Santos Alvarenga, Henrique Perini Rosa, Luiza Poppe Merigueti, Marllon Cindra Sant’Ana, Clara W.T. Koh, Raquel Furlani Rocon Braga, Rahna Gonçalves Coutinho da Cruz, Rhana Evangelista Salazar, Vinícius do Prado Ventorim, Gabriel Mendonça Santana, Thomas Erik Santos Louro, Luana Santos Louro, Flavia Imbroisi Valle Errera, Flavia de Paula, Lorena Souza Castro Altoé, Lyvia Neves Rebello Alves, Raquel Silva dos Reis Trabach, Eldamária de Vargas Wolfgramm dos Santos, Elizeu Fagundes de Carvalho, Kuan Rong Chan, Iúri Drumond Louro

**Affiliations:** a Núcleo de Genética Humana e Molecular, Departamento de Ciências Biológicas, Universidade Federal do Espírito Santo, Vitória, Espírito Santo, 29075-910, Brazil; b Departamento de Medicina Social, Universidade Federal do Espírito Santo, Vitória, Espírito Santo, 29090-040, Brazil; c Program in Emerging Infectious Diseases, Duke-NUS Medical School, 169857, Singapore; d Centro de Ciências da Saúde, Curso de Medicina, Universidade Federal do Espírito Santo (UFES), Vitória, Espírito Santo, 29090-040, Brazil; e Escola Superior de Ciências da Santa Casa de Misericórdia de Vitória (EMESCAM), Espírito Santo, Vitória, 29027-502, Brazil; f Instituto de Biologia Roberto Alcantara Gomes (IBRAG), Universidade do Estado do Rio de Janeiro (UERJ), Rio de Janeiro, 20551-030, Brazil

**Keywords:** Bioinformatics, COVID-19, Omics, SARS-CoV-2

## Abstract

Since December 2019, a new form of Severe Acute Respiratory Syndrome (SARS) has emerged worldwide, caused by SARS coronavirus 2 (SARS-CoV-2). This disease was called COVID-19 and was declared a pandemic by the World Health Organization in March 2020. Symptoms can vary from a common cold to severe pneumonia, hypoxemia, respiratory distress, and death. During this period of world stress, the medical and scientific community were able to acquire information and generate scientific data at unprecedented speed, to better understand the disease and facilitate vaccines and therapeutics development. Notably, bioinformatics tools were instrumental in decoding the viral genome and identifying critical targets for COVID-19 diagnosis and therapeutics. Through the integration of omics data, bioinformatics has also improved our understanding of disease pathogenesis and virus-host interactions, facilitating the development of targeted treatments and vaccines. Furthermore, molecular biology techniques have accelerated the design of sensitive diagnostic tests and the characterization of immune responses, paving the way for precision medicine approaches in treating COVID-19. Our analysis highlights the indispensable contributions of bioinformatics and molecular biology to the global effort against COVID-19. In this review, we aim to revise the COVID-19 features, diagnostic, prevention, treatment options, and how molecular biology, modern bioinformatic tools, and collaborations have helped combat this pandemic. An integrative literature review was performed, searching articles on several sites, including PUBMED and Google Scholar indexed in referenced databases, prioritizing articles from the last 3 years. The lessons learned from this COVID-19 pandemic will place the world in a much better position to respond to future pandemics.

## 1 Introduction

Coronaviruses (CoV) are a diverse group of positive strand RNA virus that infects many vertebrates and achieved worldwide recognition after the severe acute respiratory syndrome (SARS) outbreak in 2003. Subsequently, SARS-CoV-2 was identified to be the etiological agent of the 2019 coronavirus pandemic (COVID-19) [1]. The disease spread exponentially among countries and continents, due to the virus's high infectivity, globalization, and the speed of international traveling [2]. Pandemic rapid control has been limited by many factors, including the lack of knowledge about SARS-CoV-2 and host immunity biology, rapid diagnosis (identification of new cases), and lack of treatments. Despite all these difficulties, medical and scientific communities have been developing and sharing information at unprecedented speed, using modern bioinformatic tools to analyze big data and help establish response strategies [2,3]. Sophisticated computer models have been essential in advancing research on SARS-CoV-2. High-throughput technologies, such as Next-Generation Sequencing, and software development provided rapid identification of viral genomes that boosted advances in fighting the pandemic. In addition, bioinformatics tools provide a better characterization of severe COVID-19 immune response and better framework models for designing antiviral drugs and vaccines for this complex disease [4,5].

SARS-CoV-2 Spike protein forms a clover leaf trimer with 3 S1 heads, where the Receptor Binding Domain (RBD) is localized and an S2 trimer. The RBD binds to Angiotensin Converting Enzyme 2 (ACE2) enzymatic surface, infecting mainly bronchial ciliated epithelial cells and Type 2 Pneumocytes, whereas MERS-CoV uses dipeptidil peptidase 4 (DPP4; aka CD 26) as its receptor, and infects non-ciliated bronchial cells and type II pneumocytes [[6], [7], [8]]. In humans, ACE2 is expressed in airways epithelia, intestinal cells, lung parenchyma, vascular endothelial, neurons, astrocytes, oligodendrocytes, showing high expression in Substantia Nigra, ventricles, and middle temporal gyrus, which makes these cells susceptible to SARS-CoV-2 infection [9,10]. There are 2 possible mechanisms of SARS-CoV-2 diffusion through the hematoencephalic barrier, 1) infecting transport and vascular endothelial cells, similar to the active transport mechanism used by arboviruses and HIV to enter the central nervous system (CNS); 2) infecting immune cells that pass freely through the barriers, such as leukocytes. Considering that SARS-CoV-2 can infect lymphocytes, monocytes, granulocytes, and T-lymphocytes, this may be a plausible way to enter the CNS [9,10].

Virus transmission among humans can occur by close contact with an infected individual, especially through aerosols [10]. Studies have shown that a high percentage of infected individuals are asymptomatic, which can potentially spread to others to cause disease. Besides viral factors, how one reacts to SARS-CoV-2 infection can be defined by the host genetics, and several Whole Genome Studies (WGS) studies have tried to identify genomic loci that correlate with disease severity [11]. CRISPR studies have also revealed critical host factors that can augment SARS-CoV-2 infection. In particular, elderly individuals and those with comorbidities such as diabetes, obesity, high blood pressure, and immunosuppression, are at higher risk of developing severe disease [12]. Barek et al. [13] studied middle age and elderly patients, showing that hospitalized patients were more likely to carry comorbidities such as hypertension, diabetes, and coronary heart disease, being the most common symptoms of fever, dry cough, fatigue, dyspnea, anorexia, and respiratory discomfort. Lian et al. [8] combined genetic analysis and bioinformatics for the first time in COVID-19 research to obtain a better epidemiology understanding. Bioinformatic analysis showed 4 virus mutations and loss of amino acids in SARS-CoV-2 related to virus evolution and transmission ability. Based on these findings, World Health Organization (WHO) recommends the use of a mask covering the nose and mouth, as well as to maintain distance from infected patients [6,9,14].

Based on these brief expositions, it is observed that the application of bioinformatics and its tools, in conjunction with molecular biology, in the context of the COVID-19 pandemic, went beyond the simple identification and sequencing of the SARS-CoV-2 virus, encompassing a series of innovations and critical contributions to combating the disease. Firstly, bioinformatics techniques allowed for a detailed mapping of viral mutations, essential for tracking the virus's evolution and the rapid identification of concerning variants. Moreover, computational modeling and genomic analyses have added to the understanding of the interaction between the virus and host cells. Omics data analysis and machine learning algorithms have also provided insights into the immune responses involved in severe COVID-19 pathogenicity and protective immunity mediated by COVID-19 vaccines. Together, these studies have laid the groundwork for new targeted antiviral therapies and vaccine design. Lastly, the integration of genomics with clinical information and machine learning have allowed identification of genetic markers associated with susceptibility to infection and the severity of COVID-19, paving the way for the development of personalized treatment and prevention strategies, as well as for a deeper understanding of the molecular basis governing the varied immune responses observed among different individuals.

COVID-19 involves multiple processes and biological mechanisms, including viral evolution and variations in host responses, attributed by differences in host genetics and epigenetics. The comprehensive understanding of COVID-19 will thus require interdisciplinary approaches, including biology, mathematics, and computational sciences. This review aims to integrate the multidisciplinary bioinformatics approaches that have facilitated the better understanding of COVID-19. Articles were searched in bibliographic databases PUBMED and Google Scholar, publications in English, indexed in referenced databases and without a publication time filter, but prioritizing articles from the last 3 years, to answer the questions: (i) “What is the current knowledge about molecular biology and bioinformatics in COVID-19?”; (ii) “What are the applications of molecular biology and computational studies for understanding COVID-19 and SARS-CoV-2?”.

## 2 Diagnostic tests for SARS-CoV-2

After the first SARS-CoV-2 sequence was published in 2020, the first diagnostic tests were rapidly designed and implemented. German and American researchers designed RT-PCR tests for COVID-19 that are still in use today. USA Centers for Disease Control and Prevention (CDC) designed their COVID RT-PCR in February 2020 and started selling to the entire world [[15], [16], [17], [18]]. Production of diagnostic tests and sharing virus information among nations was fundamental for a global orchestrated response to this pandemic, which caused catastrophic outcomes and millions of lost lives around the globe [19,20]. The coronavirus nucleocapsid N protein is a structural protein required for RNA replication and has been targeted by artificial intelligence (AI) algorithms to identify immunodominant regions. Evidence suggested that protein N could thus be a potential candidate for screening SARS-CoV-2 [[21], [22], [23], [24]].

RT-PCR is the most reliable method for detecting SARS-CoV-2, offering high accuracy, and is essential for international travel [19]. Loop-mediated isothermal amplification (LAMP) is similar to PCR but uses a constant temperature (60–65 °C), making a simple, easy and rapid method for Covid detection [25,26]. CRISPR-Dx tests directly analyze nasopharyngeal samples without RNA purification, including internal controls in each reaction [27,28]. Meanwhile, the demand for instant and low-cost testing led to the widespread use of antigen rapid diagnostic tests (Ag-RDTs) during the pandemic. These tests provide immediate results, are easy to use, and require no equipment, although they are less accurate than RT-PCR [29,30]. Despite their convenience and accessibility, the preference for RT-PCR in medical settings remains due to its superior sensitivity and specificity.

This diversity of tests reflects the balance between the need for diagnostic accuracy and practicality, with bioinformatics and molecular biology playing essential roles in the optimization and ongoing innovation of these technologies to tackle COVID-19. While RT-PCR remains to be the gold standard due to its high specificity and sensitivity, with detection of viral RNA even in low concentrations, RT-PCR can be effective only in situations where the virus mutations are known. The detection of newly emerging variants will still require Whole Genome Sequencing. On the other hand, antigen tests offer the advantage of speed and simplicity, ideal for mass testing and point-of-care settings. However, the sensitivity and specificity may be influenced by the viral load and strain. Hence, the more advanced bioinformatics tools and molecular biology techniques such as metagenomics and next-generation sequencing (NGS) remain to be important for detection of new emerging variants.

For surveillance or measurement of herd immunity, serological tests which detect antibodies against SARS-CoV-2 have been employed. However, as antibody levels can wane rapidly below serological detection limits, prevalence studies that only measure antibodies as the only read-out may underestimate the extent of community exposure to the virus. Moreover, asymptomatic individuals may mount poorer antibody responses [31], which may further underestimate the true prevalence of the virus. As different SARS-CoV-2 variants can induce distinct types of antibodies, the antigenic differences among variants will also need to be considered when assessing for antibody responses [32,33].

The adaptive immune responses to SARS-CoV-2 includes both the humoral immune responses and cell-mediated immune responses. As the cell-mediated immune response is mediated by T cells, emerging studies are suggesting that SARS-CoV-2 specific T cells can be measured to evaluate previous exposure to SARS-CoV-2. The T cell responses to SARS-CoV-2 can last for more than 20 months after COVID-19 [34] and have been demonstrated to be induced early, especially in individuals who are asymptomatic or have mild disease following infection [35,36]. However, traditional T cell assays can be challenging to perform as these assays require isolation of peripheral blood mononuclear cells and require complex laboratory equipment such as flow cytometry and ELISPOT assay. Recent developments that measure T cell responses directly from whole blood have thus greatly simplified these assays without compromising the accuracy of the data, promising to provide better estimates for SARS-CoV-2 prevalence and evaluation of herd immunity.

In summary, each type of diagnostic test plays a complementary role in the overall strategy of diagnosis, tracking, and understanding of COVID-19, with the choice dependent on the clinical context, public health objectives, and availability of resources [[15], [16], [17], [18], [19], [20], [21], [22], [23], [24], [25], [26], [27], [28], [29], [30]].

## 3 Prevention and treatment

Bioinformatic tools have also played a role in disease prevention and treatment, as well as elucidating viral protein structures and ligand receptor binding sites [37]. 3DBIONOTES was created with the purpose to access quality measures and standardize information about COVID-19. Websites have been created with similar purposes, such as Coronavirus Structural Taskforce (https://github.com/thorn-lab/coronavirus_structural_task_force), helping solve viral structure and protein interactions that have an impact on disease severity [38]. Given the large amounts of data generated, AI is a helpful tool to guide COVID-19 prediction and management. Data generated by hospitals, emergency care units, and other COVID-19 healthcare registries are interpreted by AI to detect severity signs and symptoms, helping create strategies to avoid complications and mortality [[39], [40], [41]]. Deep Learning (DL) is also increasingly applied to the analysis and interpretation of Computerized Tomography, Magnetic Resonance, and X-rays, using trained algorithms that speed up the diagnosis of dangerous conditions, identify disease severity, and avoid putting health professionals at risk [42]. While the lack of diagnosis and proper patient care remains to be a problem in the developing countries, which may continue to favor disease perpetuation and the emergence of new virus strains [43], the improvements in AI promises to improve healthcare by facilitating clinical diagnosis.

### 3.1 Role of bioinformatics in the understanding of COVID-19 pathogenesis

Omics technologies have facilitated the identification of prognostic markers for determining which patients need urgent care, avoiding unnecessary hospitalization, and giving the best medical care for patients likely to evolve poorly. In the search for genetic host factors that affect patient prognosis, several genes involved in humoral immune response, interferon signaling, acute response proteins, lipid metabolism, and platelet degranulation were identified [[44], [45], [46], [47]]. Severity predictive models based on demographic and clinical variables [48,49] suggest that a combination of demographic, clinical, and gene expression measurements could in the future, serve as reliable early prognostic biomarkers for severe COVID-19, helping health professionals to provide better screening and care for critical patients (Table 1).

**Table 1:** Relevant studies that used single-cell research to help elucidate COVID-19.

Study	Reference
Computational support provided by bioinformatics enabled predictive advances and high-throughput screening to accelerate pandemics treatment and prevention, such as COVID-19, through reuse of drugs and development of new drugs and vaccines.	Ma et al. [50]
Single-cell sequencing led to deeper insights about biological systems heterogeneity, as in the case of COVID-19, providing new knowledge about virus-host cell relationships and several subpopulations and individual cell signatures have been recently better understood.	Wang et al. [51] and Cen et al. [49]
Multi omic single cell studies have shown how the immune system adapts and reacts to SARS-CoV-2, causing a viral dynamic response to decrease systemic viral load.	Unterman et al. [52], Derouiche et al. [53], Liu et al. [54], Ratnasiri et al. [55] and Wang et al. [51]
The interplay between monocytic myeloid-derived suppressor cells, neutrophils, B cells and T cells are most critical in promoting severe COVID-19 disease progression and the characterization of host responses involved in severe COVID-19 has led to host targets that can potentially be modulated to reduce disease burden.	Chan et al. [56], Ong et al. [57], Sekine et al. [58], Laing et al. [59], Kuri-Cervantes et al. [60], Hadjadj et al. [61] and Stephenson et al. [62]
In a scenario of severe COVID-19 unicellular transcriptome linked to clonal hematopoiesis of undetermined potential (CHIP), it was observed that in the presence of CHIP hyperinflammation was related to a distinct immune response of patients with severe COVID-19 progression.	Choi et al. [63]
It was identified a subpopulation of natural killer cells similar to memory cells that accumulates with aging and showed a correlation with higher COVID-19 severity. Therefore, there is a clear and unfolding relationship between COVID-19 and the immune system.	Guo et al. [64]
Single-cell RNA sequencing data from COVID-19 patients and healthy individuals treated by bioinformatics showed an interface between coagulation and immune response, through gene expression analysis, been possible to observe that protein C has a mechanistic role in the hypercoagulability syndrome that affects patients with severe COVID-19.	Silva et al. [65]
Single-cell spatial analysis created atlases of lung tissue cellular and molecular signatures from COVID-19 severe cases.	Das et al. [66]
Through ligand-receptor interaction databases and single-cell RNA sequencing (scRNA-seq), new hypotheses can be raised about intercellular signaling and cell-cell communications between different cell types.	Liu et al. [67], Qi et al. [68] and Wahiduzzaman et al. [69]
Single-cell is helping repositioning drugs and vaccine development against COVID-19, because biological networks combined with single-cell COVID-19 patients' transcriptome can reveal new gene expression signatures as theragnostic biomarkers, as in the case of post-COVID-19 pulmonary fibrosis.	Li et al. [70]
scRNA-seq helped develop protective vaccination programs and discover peripheral blood mononuclear cells useful for understanding vaccine response. And it has been providing the basis for more integrated, precise, and predictive studies together with big data to elucidate future pandemics, such as COVID-19, finding new variants and treatments.	Li et al. [71], Wang et al. [51] and Li et al. [72]

Omics technologies have also facilitated high-throughput detection and analysis of the host responses to SARS-CoV-2 infection, providing insights into the molecular mechanisms underlying severe COVID-19 pathogenesis [48]. In addition, single-cell RNA sequencing technologies have allowed deeper characterization of the immune cells and pathways involved in severe COVID-19 progression [73]. Multiomics-based methodologies have also been consistently employed to explore mechanisms involved in COVID-19's pathogenesis, potential pharmaceutical targets, and diagnostic methods. Due to their high performance, these technologies have contributed to mapping gene and protein expression profiles and understanding their intricate effects on SARS-CoV-2, as reported in various studies. These advanced omics techniques are seen as ongoing support for scientists and healthcare professionals in analyzing and comprehending the pathology of COVID-19 (Table 1) [73]. When coupled with time-series measurements, these omics signatures provide further insights on the biological pathways involved in the distinct phases of the disease, including the acute to the recovery phases of COVID-19.

For instance, Huang et al. [74] conducted an integrated bioinformatics exploration and preliminary clinical verification to identify key biomarkers in severe cases of COVID-19, concluding that the genes *MCEMP1*, *ANXA3*, *CD177*, and *SCN9A* are essential for diagnosis or development of the disease, after detecting significant changes and excessive neutrophil activation in patients, corroborated by validations in additional datasets and clinical samples. Similarly, longitudinal studies have identified neutrophil activation to track closely with severe disease progression and resolution [57,75]. These studies also highlight the advantage of using whole blood rather than the peripheral blood mononuclear cells for omics data analysis, as whole blood will allow omics characterization of the neutrophils and the other granulocyte populations. In addition, meta-analysis can provide deeper insights into the omics signatures that are most consistently represented, which may be useful for identifying predictive prognostic biomarkers. For example, by integrating and re-analysing 7 RNAseq datasets, one longitudinal study dataset and single-cell RNA-sequencing dataset collected from severe and mild COVID-19 patients, Chan et al. [56] revealed that the gene expression levels of *MCEMP1* and *HLA-DRA* in CD14^+^ cells could serve as potential early prognostic indicators for severe disease progression. The study also pinpointed the role of myeloid-derived suppressor cells in facilitating severe COVID-19 progression, which is increasingly supported by other studies [76]. Given the severity predictive models based on demographic and clinical variables [77], we envisage that a combination of demographic, clinical and gene expression measurements could in future, serve as reliable early prognostic biomarkers for severe COVID-19, helping health professionals to better triage and provide better care for the critical patients (Table 1).

The integration of multi-omic studies from COVID-19 patients with the other known diseases can also offer insights into the biological processes involved in severe COVID-19. For instance, it has been suggested that the omics signatures for severe COVID-19 shares with the non-alcoholic fatty liver disease (NAFLD), highlighting that some of the pathophysiological mechanisms between COVID-19 and severe metabolic syndrome could be shared [78]. In addition, comparing the gene expression signatures between bacterial sepsis and severe COVID-19 have provided insights into the roles of myeloid suppressive cells in modulating disease severity in both bacterial sepsis and severe COVID-19 [79]. Bioinformatics algorithms are also now helping to identify signatures related to post-COVID sequelae and Long COVID symptoms. For example, quantitative immunological scores can stratify patients according to therapy response, allowing for precise classification of patients based on the host immune response [80,81]. Furthermore, the use of multi-omic approaches have also identified potential factors related to adverse events arising from mRNA vaccines against COVID-19. For instance, by integrating gene expression and cytokine data, Ong et al. [57] revealed RNASE2 as a potential trigger of increased inflammation, where the increased expression of baseline RNASE2 in an individual resulted in heightened levels of Th2 cytokines, pro-inflammatory cytokines and inflammation-induced growth factors after mRNA vaccination, giving rise to occurrence of a rare cardiac side effect [57].

At present, the main challenge in multiomics analysis is to integrate AI to perform multimodal, multifactorial data analysis and develop a dependable clinical trajectory model for clinical predictors of disease severity, prevention, and treatment [54,82,83]. To execute machine learning and AI effectively, there is a need to gather, process, and consolidate copious amounts of data systematically. In addition, while pathway enrichment tools are extensively researched for transcriptomics, our knowledge of metabolomics, epigenetics, and methylation remains poor, posing challenges in integrating different omics results [84]. At present, supervised models have provided more insights on the omics results as they are more straightforward to implement based on the conditions applied. However, several other artificial intelligence models which are unsupervised, including the generative transformer-based models and linked entity relationship models can hold great promise in the future to predict clinical disease trajectories based on omics and clinical data. We thus believe that the future development of more sophisticated machine learning and AI algorithms will continue to be instrumental in reducing the dimensionality of the multi-omics data and facilitate data analysis.

### 3.2 Role of bioinformatics in vaccine development

Bioinformatics has played a major role in the development of vaccines for SARS-CoV-2 (Table 2) [85,86], facilitated by the growing availability of curated online data repositories [87]. There are four major strategies that have been considered for SARS-CoV-2 vaccine design: 1) complete virus vaccines, making use of inactivated or attenuated viruses, but modifying the exoribonuclease and spike protein genes to reduce virulence and replication capacity. These induce a holistic adaptive immune response to the virus as most of the viral proteins are given; 2) Recombinant RNA vaccines, commonly used to increase vaccine safety by using only specific immunogenic viral proteins, without the need to inoculate the whole virus during vaccination. The most used viral proteins are the Spike protein (S), Nucleocapsid protein (N) and membrane protein (M); 3) Virus Like Particles Vaccines and 4) mRNA vaccine [88,89].

**Table 2:** Bioinformatics tools used to create vaccines against SARS-CoV-2.

Tools	Description	Function in facing COVID-19	Access Link
modlAMP	Python package that works with peptides, proteins, and amino acid sequences. Provides a rapid capability to generate and evaluate the efficacy of peptides but requires extensive experimental validation of the proposed peptides.	Identification of peptide bonds with inhibitory potential against SARS-CoV-2.	https://modlamp.org/index.html
PEP-FOLD3	Predict amino acid sequences. Highlights the ability to predict how peptides fold and interact with the virus. However, the accuracy of the predictions can vary, especially for larger peptides or those with complex structures.	Peptide modelling, 3D structures of human immune cell epitopes.	https://bioserv.rpbs.univ-paris-diderot.fr/services/PEP-FOLD3/
IEDB	Group of tools used to characterize experimentally identified epitopes. Indispensable for understanding the immune response to SARS-CoV-2. However, interpreting the data requires a deep knowledge of immunology, and constant updating of the database is crucial to maintain its relevance.	Prediction and validation of SARS-CoV-2 epitopes.	http://tools.iedb.org/main/
DiscoTope	B-cell epitope prediction using 3D protein modeling. Assists in identifying regions of the virus that are more likely to be recognized by antibodies. However, its applicability is restricted, leaving aside other aspects of the immune response.	Identification of B-cell epitopes in SARS-CoV-2 viral proteins.	http://www.cbs.dtu.dk/munent/DiscoTope-2.0/
ToxinPred	*In silico* method for prediction of toxic protein domains. Useful in ensuring that developed peptides are not toxic. However, toxicity prediction is not always accurate and may require experimental confirmation.	Toxicity prediction of candidate COVID-19 vaccines.	http://crdd.osdd.net/munen/toxinpred/
SVMTriP	Realistic prediction of surface protein antigenic epitopes. Capable of predicting epitopes with a strong immune response. However, its effectiveness depends on the quality and quantity of training data.	Identification of antigenic epitopes to help design new vaccines.	http://sysbio.unl.edu/SVTriP/
AlphaFold	AI system that predicts the 3D structure of proteins from the amino acid sequence. Has huge implications in understanding the viral machinery and developing therapies. However, it requires extensive computational resources.	Modeling of SARS-CoV-2 Spike Protein	https://alphafold.ebi.ac.uk/

*Adapted from Chukwudozie et al. [85].

Immunoinformatic applied bioinformatics to immunology, involving complete analysis of an organism's genes and proteins involved in the maturation of the immune system to predict the immune response against specific pathogens or molecules [79,88]. This method has been applied to determine how the immune system reacts to COVID-19, and to predict the most immunogenic SARS-CoV-2 regions [85,88,89]. This information is important as animal models may not accurately reflect the reactogenicity and immunogenicity of vaccines. Bioinformatics commonly used tools are: TEpredict, CTLPred, NetMHC and Epitopemap [85]. Furthermore, Reverse Vaccinology (RV) is a process that uses AI to identify promising virus antigens to start developing new vaccines. This method uses pathogen genome information, coupled with available proteomic and transcriptomic data to find surface proteins that play a role in the host pathogen internalization [57,85]. This process was successful in designing new vaccines for Serogroup B Meningococcal Disease, leading to the approval of Bexsero® vaccine [57]. The most notable advantages of RV are faster timeframe and less cost for achieving new vaccines, mainly due to reducing the number of candidate proteins and making use of antigens expressed in low amounts or only in a specific period of the pathogen life cycle. This technology has been used to develop multiepitope chimeric vaccines against SARS-CoV-2, making use of a vaccine design tool called Vaxign to predict candidate RV vaccines against diverse viruses and bacteria [57,85].

While bioinformatics tools have advanced COVID-19 vaccine development, their integration into conventional vaccine creation faces challenges (Table 2). The RNA-based nature of the novel coronavirus, unlike the stable genomes of DNA viruses, increases its mutation rate, which may compromise vaccine effectiveness. Overcoming these issues is crucial to increasing the safety and effectiveness of vaccines against SARS-COV-2 and other new viruses. Despite these limitations, these technologies have guided vaccine development by improving antigen design to make vaccines with better safety and immunogenicity profiles [87,88,90].

As shown in Tables 1 and it is highlighting three fronts of action: i) Predictive Power: with an emphasis on AlphaFold for its ability to predict protein structures with high precision. Notably, other tools like PEP-FOLD3 can be applied for peptide modeling, though AlphaFold surpasses it in terms of precision and scope; ii) Applicability in therapies: modlAMP and ToxinPred offer promising pathways for the development of therapies, focusing on antimicrobial peptides and toxicity, respectively. However, the need for experimental validation is a common limitation; and iii) Vaccine design: IEDB, DiscoTope, and SVMTriP are essential for vaccine design, each focusing on aspects of immune response. IEDB offers a comprehensive view of epitopes, while DiscoTope and SVMTriP provide insights into B cell epitopes and immunogenicity, respectively [85].

### 3.3 Role of bioinformatics in drug design

During the initial year of the pandemic, as the world grappled with a severe global health crisis, there was a rapid advancement in the development of computational tools aimed at drug discovery (Table 3). In particular, AI models have transformed the landscape of drug discovery, with studies underscoring their profound influence. Traditional drug discovery processes are known for their excessive costs and extensive timelines. Computational methodologies are thus pivotal in identifying drug targets and even discovering overlooked drug candidates [91], pioneering a novel medical domain for utilization during worldwide emergencies such as the COVID-19 pandemic [92]. Numerous other studies have described unique advances in drug research against COVID-19 through the construction of various computational tools (Table 3).

**Table 3:** Important studies that used bioinformatics in new drug design and discovery.

Study	Reference
Bioinformatics was used to elucidate the 3D structure of SARS-CoV-2 protease, to predict molecular docking and to simulate drug safety, all of which were essential for developing drugs against COVID-19.	Chukwudozie et al. [85]
Computer Aided Drug Design (CADD) rely upon Structure Based Drug Design (SBDD) and Ligand Based Drug Design (LBDD), both of which need high performance algorithms to model realistic interactions among designed molecules with host and viral ones.	Selvaraj et al. [93]
Putative signaling pathways able to modulate infection reduction, due to decreased viral replication and less symptoms associated with SARS-CoV-2, with help of an application programing interface (API), predicting efficacy of a dozen existing human approved drugs, among which drugs used to treat Systemic Lupus Erythematosus (LES) and Multiple Sclerosis (MS).	Scott et al. [90]
Virtual screening along with *in silico* predictions and molecular docking analysis provided two possible candidate antiviral drugs for COVID-19 and it was possible to simulate patterns of absorption, distribution, metabolism, and toxicity (ADME) for drugs against SARS-CoV-2.	Ghaebi et al. [94], Hage-Melim et al. [95], Rahman et al. [96] and Alghamdi et al. [97]
Molecular docking algorithms such as GLIDE, AutoDock Vina and SwissDock, focusing on interactions between drug molecules and viral proteins using 3D modeling, provide the opportunity to find existing drugs with high affinity for viral proteins, followed by use of other bioinformatics tools to reduce the number of candidate molecules.	Aronskyy et al. [98] and Chaudhari et al. [99]
*In silico* docking studies have shown that Remdesivir has multiple potential protease inhibition (PLpro) sites and molecular modeling of all SARS-CoV-2 protein structures using homology modeling suggests several drugs as potential antiviral agents, antibiotics, and muscle relaxants.	Wu et al. [100], Sakr et al. [101], Eweas et al. [102] and Frances-Monerris et al. [103]
Many computational tools were rapidly created in face of the mortal danger the world population was facing during the pandemic's first year and Deep Learning based Ligand Design predicts poly pharmacologic profiles in human proteins and putative viral drug targets. Resulting data were stored in PolypharmDB, allowing for future discovery of multiple candidate drugs for COVID-19.	Waman et al. [92]
Computational approaches have directed clues to identify drug candidates that were previously overlooked, giving rise to a crucial new medical field for global emergencies such as the COVID-19 pandemic.	Kumar et al. [104] and Ma et al. [50]

Bioinformatics was used to elucidate the 3D structure of SARS-CoV-2 protease, to predict molecular docking and to simulate drug safety, all of which were essential for developing drugs against COVID-19 [85]. Databank repositories such as D3Targets-2019-nCoV, CoViLigands, CORDITE, DockCov2, DrugBank, TCMD, together with AI algorithms (DNN, MolAICal) and molecular coupling softwares (AutoDock, D3Docking, D3Similarity, CDOCKER) created a useful platform to predict effectiveness and safety of potential drugs against SARS-CoV-2, as well as repurposing existing drugs (Table 4) [50]. Through the application of molecular docking analysis for virtual triage of SARS-CoV-2's main protease protein (Mpro), Moradi et al. [91] discovered two potential antiviral drugs for combating COVID-19, a finding further elaborated on by Waman et al. [92]. In parallel, Mirza and Froyen [105] and Ghaebi et al. [94] utilized *in silico* techniques to model the ADME profiles of drugs targeting SARS-CoV-2, contributing valuable insights into their pharmacokinetics. Techniques based on DL for designing ligands were able to predict interactions with human proteins and theoretical viral drug targets, with the compiled data stored in PolypharmDB. The High Ambiguity Driven protein-protein DOCKing (HADDOCK) web server facilitated the preliminary selection of drugs to fight SARS-CoV-2 by examining the interactions among already identified proteins and speculative molecular ligands [92].

**Table 4:** Computational tools used to prospect new drugs in the current pandemic.

Tool	Description	Access Link
D3Targets-2019-nCoV	Web server for the identification of potential antiviral agents against SARS-CoV-2.	https://www.d3pharma.com/D3Targets-2019-nCoV/index.php
CoViLigands	Database of anti- SARS-CoV-2 molecules.	https://www.d3pharma.com/D3Targets-2019-nCoV/CoViLigands/2019-nCoV.php
DockCoV2	Predicts drug affinity with SARS-CoV-2	https://covirus.cc/drogas/
CORDITE	Database of potential anti- SARS-CoV-2 drugs.	https://cordite.mathematik.uni-marburg.de
DRUGBANK	Database of drugs and potential targets.	http://redpoll.pharmacy.ualberta.ca/drugbank/index.html
TCMD	Chinese traditional medicine database.	https://www.drugbank.ca
AutoDock	Bioinformatics tool set for prediction of receptor-ligand interactions.	http://autodock.scripps.edu/
D3Similarity	*In silico* prediction of target proteins for experimental molecules.	https://www.d3pharma.com/D3Targets-2019-nCoV/D3Similarity/index.php
ESP DNN	Deep Neural Networks tool used for drug discovery	https://github.com/AstexUK/ESP_DNN
AlphaFold	AI system that predicts the 3D structure of proteins from the amino acid sequence.	https://alphafold.ebi.ac.uk/

*Adapted from Chukwudozie et al. [85] and Ma et al. [50].

Computer Aided Drug Design (CADD) rely upon Structure Based Drug Design (SBDD) and Ligand Based Drug Design (LBDD), both of which need high performance algorithms to model realistic interactions among designed molecules with host and viral ones [93]. Coronavirus Explorer (CoVex) is another tool used to predict drug-protein interactions, as well as SARS-CoV-2 and human protein interactions, predicting efficacy of new and existing drugs [85]. Moradi et al. [91] used virtual triage of the main SARS-CoV-2 protease protein (Mpro) by means of molecular docking analysis and found two potential candidate antiviral drugs for COVID-19. *In silico* predictions were also used by Mirza and Froyen [105] and Ghaebi et al. (2020) [94] to simulate patterns of absorption, distribution, metabolism, and toxicity (ADME) for drugs against SARS-CoV-2 (Table 4).

Molecular docking algorithms, such as GLIDE, AutoDock Vina e SwissDock, focused on interactions between drug molecules and viral proteins using 3D modeling, in order to find existing drugs with high affinity for viral proteins [99], followed by other bioinformatic tools to narrow down the number of candidate molecules [98]. Wu et al. [100] and Sakr et al. [101] molecular modeled all SARS-CoV-2 protein structures using modeling by homology. These models suggested several drugs as potential antiviral agents, antibiotics, and muscle relaxants. Sakr et al. [101] used *in silico* docking studies to show that Remdesivir has multiple potential protease inhibitory sites (PLpro). These computational methodologies are pivotal in discovering overlooked drug candidates [85], pioneering a novel medical domain for utilization during worldwide emergencies such as the COVID-19 pandemic (Table 4) [85].

In addition, besides targeting the virus directly, another approach could be to target the host factors involved in severe COVID-19 pathogenesis. Using an Application Programming Interface (API), Scott et al. [90] were able to identify potential signaling pathways that could lower infection rates through reduced viral replication and diminished symptoms of SARS-CoV-2. Ebrahimi and Roshani [106] presented a comprehensive study that aims to identify key genes and potential drug combinations to treat COVID -19 through systems biology approaches. The study used a variety of bioinformatics techniques to analyze genes related to COVID-19, construct and analyze protein-protein interaction (PPI) networks, examine the controllability of signaling pathways, and identify potential therapeutic drugs based on gene-drug interactions. Meanwhile, the EXSCALATE4COV consortium focused on uncovering new therapeutic methods and promising virtual trial candidates, with their findings logged in repositories like ChEMBL [107]. Alongside the aid of two other specialized learning databases, these techniques played a key role in pinpointing drugs that can be used against COVID-19 [108]. This effort highlighted 13 potential drugs (including bedaquiline, brequinar, and celecoxib) as key to halting virus replication, paving the way for novel antiviral treatment developments [108]. In summary, using drug-gene interaction databases, the study proposed combinations of drugs that could target genes involved in the pathogenesis of COVID-19. This approach suggests new uses for existing medicines (drug repurposing) as potential treatments for COVID-19.

In summary, what stands out with such tools presented in Table 3, Table 4 are applications in: i) Precision and applicability: having AlphaFold with its precision and transformative potential in understanding protein structures, providing a solid foundation for the design of new drugs. Moreover, AutoDock is known for its remarkable validity and widespread use in docking simulation; ii) Agility in drug discovery: D3Targets-2019-nCoV, D3Similarity, and CoViLigands being essential for the rapid identification of targets and compounds, accelerating the initial phase of drug discovery; and iii) Integration of traditional and modern data: having TCMD and DRUGBANK exemplify how traditional knowledge and extensive databases can be valued and utilized in the fight against COVID-19.

## 4 The need to collaborate and equip researchers with bioinformatics skills

Close collaborations between virologists, immunologists, mathematicians, and computational scientists have facilitated the use of bioinformatics in vaccine and drug development, as well as a deeper understanding of the molecular mechanisms underpinning COVID-19 pathogenesis. In addition, there is a need to encourage researchers to be multidisciplinary, to apply AI, ML, neural networks and DL software packages to solve research questions in virology, biology, biochemistry, genetics and molecular biology. These competences also allow researchers to develop integrative complex codes as tools to help in deeper understanding of SARS-CoV2 genomic variability, virus-receptor interactions, molecular docking, prognosis prediction, vaccine design and candidate drugs for treatment [50,109].

Given the vital role of bioinformatics in the COVID-19 pandemic, it is likely that bioinformatics will play a critical role in future outbreaks or pandemics. There is thus a critical need to train and equip more individuals with bioinformatics skills. This knowledge gap may be more prominent in certain populational subgroups depending on socioeconomical conditions, where countries and continents such as Africa, Asia and Latin America have limited expertise, although the situation is slowly improving over the recent years [110]. Dissemination of bioinformatics knowledge through trainings and workshops should be highly encouraged, and some specific initiatives are succeeding in this goal, including initiatives developed by Systems Biology Laboratory of the Oswaldo Cruz Foundation (FIOCRUZ) [111].

## 5 Perspectives and applications

Throughout this review, it can be understood that the COVID-19 pandemic has provided prominent highlights regarding technological advances in science as a whole, especially in terms of viral immunology permeating the computational area, new diagnostic, preventive, and treatment approaches that are guided by new trends in precision medicine, and additionally, development of machine learning and AI tools to understand big data from clinical trials and human patients. It is also noteworthy to consolidate the advancements in bioinformatics as a result of the COVID-19 pandemic to prepare for the next one, and witness how bioinformatics has promoted social, scientific, and economic innovation on a global scale.

Computational analyses have facilitated the understanding of the relationships with COVID-19 and new therapeutic approaches or applications to the treatment sector, providing fresh insights on the genes and networks associated with COVID-19, and highlighting central genes, potential compounds, and drugs that can be used against SARS-CoV-2 [112].

Furthermore, the inclusion of multi-omics and single-cell data has demonstrated their importance in the identification and prediction of COVID-19 disease phenotypes, including the use of machine learning and deep learning methods that have significantly advanced disease treatment and prevention [113]. Moreover, structure prediction and molecular dynamics simulations provided insights into drug and vaccine design. Better computing infrastructure have also allowed big data storage and execution of complex computations and data processing tasks related to protein structure predictions, protein docking, omics data analysis and AI [114].

The increasing focus on working with vast datasets in a brief time throughout the pandemic supported a pioneering move towards a bioinformatics adept at preventive medicine in screening and early detection, and in epidemiology driven by enormous amounts of global research outcomes [115]. The realms of AI have advanced in creating methods and services for combating the disease and structured opportunities and perspectives that are multidisciplinary and collaborative, as well as a formalized socioeconomic trend as a pillar of research [116].

**Fig. 1:**
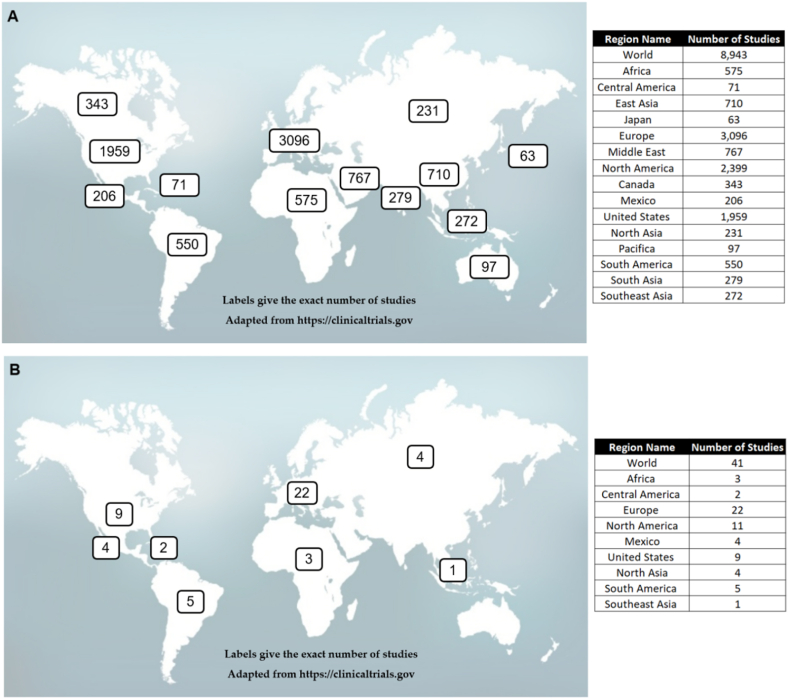
Global distribution of COVID-19 clinical trials. Clinical studies with specific keywords are shown: “COVID-19” alone (a) and “bioinformatics” or “computational biology” and “COVID-19” (b). Despite the substantial number of clinical studies on COVID-19, studies addressing bioinformatics/computational biology are still scarce.

Moreover, in terms of clinical aspects, as we described earlier, the potential applications of bioinformatics for the study of COVID-19 (strengthened by the advancement of high-throughput technologies) have shown great promise in this field, revolutionizing our knowledge about biological mechanisms associated with COVID-19, although there are still several obstacles to the implementation of bioinformatics in clinical practice. In the future, computational approach tools will be a key element to speed up the diagnosis, treatment, and cure of COVID-19. Expanding COVID-19 computational models will contribute to the understanding of this complex disease. To research potential COVID-19 studies worldwide, we searched https://clinicaltrials.gov (accessed on April 21, 2023) and found 8943 studies for “COVID-19” alone. However, when we used the terms “bioinformatics” or “computational biology” with the term “COVID-19,” we found only 41 studies. Fig. 1a shows the distribution of COVID-19 clinical trials around the globe, and Fig. 1b shows COVID-19 with the terms “bioinformatics” or “computational biology”. Studies with no location are not included in the counts or on the map, and studies with multiple locations are included in all corresponding regions.

**Fig. 2:**
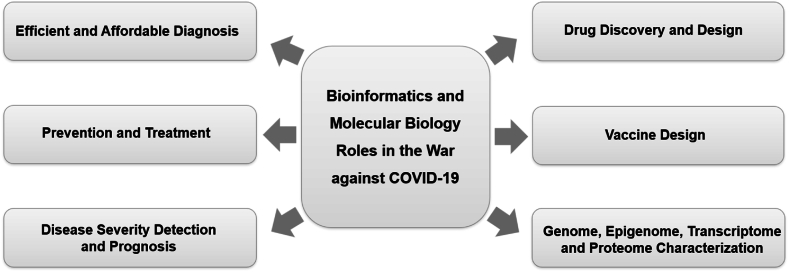
Bioinformatics and Molecular Biology Roles in COVID-19 pandemic.

While COVID-19 has caused significant mortality and morbidity worldwide, it has also tightened the social bonds with science, which reverberated on the development of new public policies, health education programs, big data analysis, evaluation of social determinants, the importance of continuous surveillance, the influence of non-viral factors, and the integration with electronic health records (EHRs) to understand more about the SARS-CoV-2 virus and its variants, achieved through rigorous computational planning with modeling techniques and machine learning algorithms [109,[117], [118], [119], [120], [121]]. The convergence of different fields of omics, sequencing, and others with the computational area of machine learning, focusing on the transparency and interpretability of methods, data sharing, and greater equity and inclusion have allowed updates to be shared worldwide, allowing re-analysis and consolidation of data to gain a more comprehensive understanding about COVID-19 [[122], [123], [124]]. The outline on the roles of bioinformatics in our understanding of COVID-19 is as summarized in Fig. 2.

## 6 Conclusion

In this review, we describe how bioinformatics helped combat COVID-19 on many fronts such as an efficient and affordable diagnosis, prevention and treatment, drug discovery and design, genome, epigenome, transcriptome and proteome characterization, disease severity detection, prognosis, and vaccine design. Many molecular biology techniques have assisted in the understanding of COVID-19, which have facilitated vaccine and therapeutics development. Some of these include Next Generation Sequencing, Metagenomics, RNAseq, Single Cell studies, Whole Genome Sequencing, Genome-Wide Association Studies, and Real-Time Polymerase Chain Reaction, allowing for rapid discovery of viral genome sequence and structure, identifying emerging virus strains, as well as susceptibility markers, disease severity, and candidate viral protein targets. We believe that these bioinformatics tools and lessons learned from the COVID-19 pandemic will remain relevant for managing the next pandemic, which will facilitate the development of vaccines and therapeutics to reduce disease burden worldwide. Research, as referenced by numerous authors, has led to quick progress in disease management and understanding, including advancements in predictability, pathogenesis, clinical management, and the application of technology and data science to combat the pandemic. Future research on SARS-CoV-2 and severe COVID-19 will increasingly demand integration and multidisciplinary due to the large amount of data, which could achieve improvements in therapeutic personalization that are better adapted to patients' adversities and predictions. To accelerate developments in these aspects, more emphasis will be needed to highlight the importance of bioinformatics in research and more resources should be provided to equip and nurture more scientists with the bioinformatics skills. We believe that the synergy and collaboration of bioinformatics with the other broad disciplines will not only allow for the identification of new and more specific therapeutic targets, but also the understanding of viral evolution mechanisms and immune escape, contributing to more effective prevention and control of COVID-19 and future pandemics.

## Data availability statement

No data was used for the research described in the article.

## Funding

This research was supported by the Individual Research Grant (MOH-000610).

## CRediT authorship contribution statement

**Débora Dummer Meira:** Conceptualization, Data curation, Formal analysis, Methodology, Supervision, Writing – original draft, Writing – review & editing. **Aléxia Stefani Siqueira Zetum:** Conceptualization, Methodology, Writing – original draft, Writing – review & editing. **Matheus Correia Casotti:** Conceptualization, Formal analysis, Methodology, Supervision, Writing – original draft, Writing – review & editing. **Danielle Ribeiro Campos da Silva:** Conceptualization, Writing – original draft, Writing – review & editing. **Bruno Cancian de Araújo:** Writing – original draft, Writing – review & editing. **Creuza Rachel Vicente:** Writing – original draft, Writing – review & editing. **Daniel de Almeida Duque:** Writing – original draft, Writing – review & editing. **Bianca Paulino Campanharo:** Writing – original draft, Writing – review & editing. **Fernanda Mariano Garcia:** Writing – original draft, Writing – review & editing. **Camilly Victória Campanharo:** Writing – original draft, Writing – review & editing. **Carla Carvalho Aguiar:** Writing – original draft, Writing – review & editing. **Carolina de Aquino Lapa:** Writing – original draft, Writing – review & editing. **Flávio dos Santos Alvarenga:** Writing – original draft, Writing – review & editing. **Henrique Perini Rosa:** Writing – original draft, Writing – review & editing. **Luiza Poppe Merigueti:** Writing – original draft, Writing – review & editing. **Marllon Cindra Sant’Ana:** Writing – original draft, Writing – review & editing. **Clara W.T. Koh:** Writing – original draft, Writing – review & editing. **Raquel Furlani Rocon Braga:** Writing – original draft, Writing – review & editing. **Rahna Gonçalves Coutinho da Cruz:** Writing – original draft, Writing – review & editing. **Rhana Evangelista Salazar:** Writing – original draft, Writing – review & editing. **Vinícius do Prado Ventorim:** Writing – original draft, Writing – review & editing. **Gabriel Mendonça Santana:** Writing – original draft, Writing – review & editing. **Thomas Erik Santos Louro:** Writing – original draft, Writing – review & editing. **Luana Santos Louro:** Writing – original draft, Writing – review & editing. **Flavia Imbroisi Valle Errera:** Writing – original draft, Writing – review & editing. **Flavia de Paula:** Writing – original draft, Writing – review & editing. **Lorena Souza Castro Altoé:** Supervision, Writing – review & editing. **Lyvia Neves Rebello Alves:** Writing – original draft, Writing – review & editing. **Raquel Silva dos Reis Trabach:** Writing – original draft, Writing – review & editing. **Eldamária de Vargas Wolfgramm dos Santos:** Conceptualization, Writing – original draft, Writing – review & editing. **Elizeu Fagundes de Carvalho:** Conceptualization, Methodology, Writing – original draft, Writing – review & editing. **Kuan Rong Chan:** Conceptualization, Funding acquisition, Supervision, Writing – original draft, Writing – review & editing. **Iúri Drumond Louro:** Conceptualization, Writing – original draft, Writing – review & editing.

## Declaration of competing interest

The authors declare that they have no known competing financial interests or personal relationships that could have appeared to influence the work reported in this paper.
